# Cervical Motor and Nociceptive Dysfunction After an Acute Whiplash Injury and the Association With Long-Term Non-Recovery: Revisiting a One-Year Prospective Cohort With Ankle Injured Controls

**DOI:** 10.3389/fpain.2022.906638

**Published:** 2022-07-07

**Authors:** Helge Kasch, Tina Carstensen, Sophie Lykkegaard Ravn, Tonny Elmose Andersen, Lisbeth Frostholm

**Affiliations:** ^1^Department of Neurology, Aarhus University Hospital, Aarhus, Denmark; ^2^Department of Clinical Medicine, Aarhus University, Aarhus, Denmark; ^3^Department of Functional Disorders and Psychosomatics, Aarhus University Hospital, Aarhus, Denmark; ^4^Specialized Hospital for Polio and Accident Victims, Roedovre, Denmark; ^5^Department of Psychology, University of Southern Denmark, Odense, Denmark

**Keywords:** acute whiplash injury, prospective observational study, nociceptive dysfunction, motor dysfunction, control group

## Abstract

**Aims:**

To explore the development of cervical motor and nociceptive dysfunction in patients with whiplash (WPs) and non-recovery based on injury-related work disability 1-year after injury when compared with ankle-injured controls (ACs).

**Methods:**

A 1-year observational prospective study examining consecutive WPs and age- and sex-matched ACs at 1 week,3 months, 6 months, and 1 year post-injury using semi-structured interviews; global pain rating (VAS0-10) and the pain rating index (PRI-T) and number-of-words-chosen (NWC) from the McGill Pain Questionnaire; examining nociceptive functioning using the cold pressor test (CPT), pressure algometry, and methodic palpation, and central pain processing using counter-stimulation; and examining motor functioning by active cervical range-of-motion (CROM), and neck strength [maximal voluntary contraction flexion/extension (MVC)]. One-year work disability/non-recovery was determined using a semi-structured interview.

**Results:**

A total of 141 WPs and 40 ACs were included. Total pain rating index (PRI-T) NWC were higher in ACs after 1 week but higher in WPs after 3 months, 6 months, and 1 year. Ongoing global pain was higher in WPs after 1 week and after 3 and 6 months but not after 1 year. Pressure pain thresholds were reduced, and palpation was higher in the neck and jaw in WPs after 1 week but was not consistently different afterward from ACs. Cervical mobility was reduced in WPs after 1 week, 3 months, and 6 months but not after 1 year, and MVC was significantly reduced in WPs when compared with ACs after 1 week and 1 year but not after 3 and 6 months. One-year non-recovery was only encountered in 11 WPs and not in the AC group. Non-recovered WPs (N-WPs) had consistently significantly higher VAS_0−10_, PRI-T, NWC, reduced pressure pain thresholds, raised muscle-tenderness, reduced active cervical range-of-motion, reduced active-neck-flexion/extension, and reported higher neck disability scores than recovered WPs. Of special interest, there was increasing tenderness in trigeminal-derived muscles based on palpation scores, and marked reduction of PPDT was most pronounced in N-WPs when compared with recovered WPs and ACs.

**Conclusion:**

Cervical motor dysfunction and segmental nociceptive sensitization were present from early after injury in WPs and prolonged in N-WPs. Differences in trigeminal and cervical motor and sensory function in N-WPs could be of interest for future treatment studies.

## Introduction

The consequences of acute whiplash are recognized, and the development of the whiplash-associated disorder (WAD) remains of major concern (1–3). A recent focus on the development of WAD has been on biopsychosocial factors, including the role of nociceptive dynamics. Given that the pain response may be altered after stressful accidents (4, 5) and injuries (6), it has further been speculated whether long-term nociceptive dysfunction is due to peripheral, segmental, or central nociceptive sensitization. Including control groups exposed to other types of minor injuries resembling whiplash injuries could therefore be crucial for examining the role of whiplash injuries in the development of long-term disabling conditions.

The present study was a *post-hoc* examination of a previously published 1-year prospective study of acute whiplash patients (WPs) and acute non-sport ankle-injured controls (ACs). The present study aimed to further examine pain reporting, the development of nociceptive function in both trigeminal and non-trigeminal derived muscles and active cervical motor function after injury, and the development of peripheral, segmental, and central nociceptive control. The primary outcome measure was 1-year work disability. Specifically, we explored three hypotheses:

First, we hypothesized that active cervical motor dysfunction (defined as reduced active neck mobility and reduced active isometric neck flexion and extension) was encountered after an acute whiplash injury but not in a control group with injuries remote from the neck. Second, we hypothesized that recovered WPs and non-recovered WPs (N-WPs) would differ significantly in early active cervical motor dysfunction and nociceptive dysfunction. Third, we hypothesized that neck-related and jaw-related tenderness would differ significantly between WP and AC groups and that trigeminal involvement would play a role in non-recovery.

## Materials and Methods

### Study Design

The study was an observational prospective study examining consecutive acute WP exposed to rear-end motor vehicle accidents and a matched control group exposed to acute non-sport ankle distortion.

### Study Participants

During a 1-year inclusion period from January 1997 to January 1998, consecutive WPs and ACs seen at emergency units in Aarhus County were invited to participate.

The WP inclusion criteria were as follows: exposure to rear-end car collision, preservation of full consciousness during a collision, no sign of amnesia after the injury, contact to the local emergency unit within 2 days after collision presenting with whiplash-related complaints (neck pain, headache, and stiffness of neck), and age ranging from 18 to 70 years (WAD grades I, II, III). The AC inclusion criterion was exposure to non-sport ankle distortion.

The exclusion criteria for both WP and AC groups were as follows: previously known considerable neck or back disorder; previous significant posttraumatic headache complaints; known medical history of severe headache, migraine, or widespread pain; a record of considerable psychiatric disorder; and known medical or alcohol abuse. An x-ray verified the findings of fractures and dislocations.

Participants provided informed written and verbal consents and participated in semi-structured interviews on previous and present medical records.

Details of control group participants: To form a gender- and age-matched control group, a group of 40 out of the first 100 consecutively included WPs were randomly selected (21 women and 19 men) and subsequently pairwise-matched with consecutive ACs, fulfilling the inclusion criteria and belonging to the same age group within the seven age groups categorized (18–24, 25–29, 30–34, 35–39, 40–49, 50–59, and 60–67 years) and the same sex.

### Study Procedure

During a 1-year inclusion period, consecutive WPs and ACs seen at one of two emergency units in Aarhus County were invited to participate. Eligible participants provided informed written and verbal consents before participating in the data collection. Data collection methods were semi-structured interviews, questionnaires, and clinical tests. These were repeated at each time point (7). All examinations were done by the same examiner (HK). The examiner was not blinded when examining the participants belonging to the WP or the AC group. Non-recovery status was determined after all examinations had been done.

### Study Variables

Several different variables were included in the present study. At baseline, indicators of cervical motor dysfunction and nociceptive dysfunction were measured as tests for cervical motor dysfunction, active cervical range of motion (CROM), neck strength extension/flexion nociceptive dysfunction, pressure algometry, and methodic palpation, and CPT was performed. In addition to this, participants completed the McGill Pain Questionnaire (MPQ) for assessment of nociceptive dysfunction. These tests and variables are described further below.

Of note, patients reported the use of medical and non-medical treatment for their relevant injury (WP/AC) during the 1-year observation period; these data are presented elsewhere (8).

### McGill Pain Questionnaire

The MPQ is a validated self-report instrument for assessing the quality and intensity of pain (9). In the present study, participants were asked about their current global pain (ongoing self-reported pain, VAS_0−10_). The Danish version of MPQ comprises 78 words divided into 20 groups, of which the respondent chooses the words best describing the experience of pain symptoms. Afterward, the reported pain is categorized into 5 pain rating indices, including PRI-S (sensory), PRI-A (affective), PRI-E (evaluative), PRI-M (miscellaneous), and PRI-T (total). The number of words chosen (NWC) ranges from 0 to 20, with higher numbers indicating pain describing words from several word groups and categories.

### Pressure Algometry

**Figure 1:**
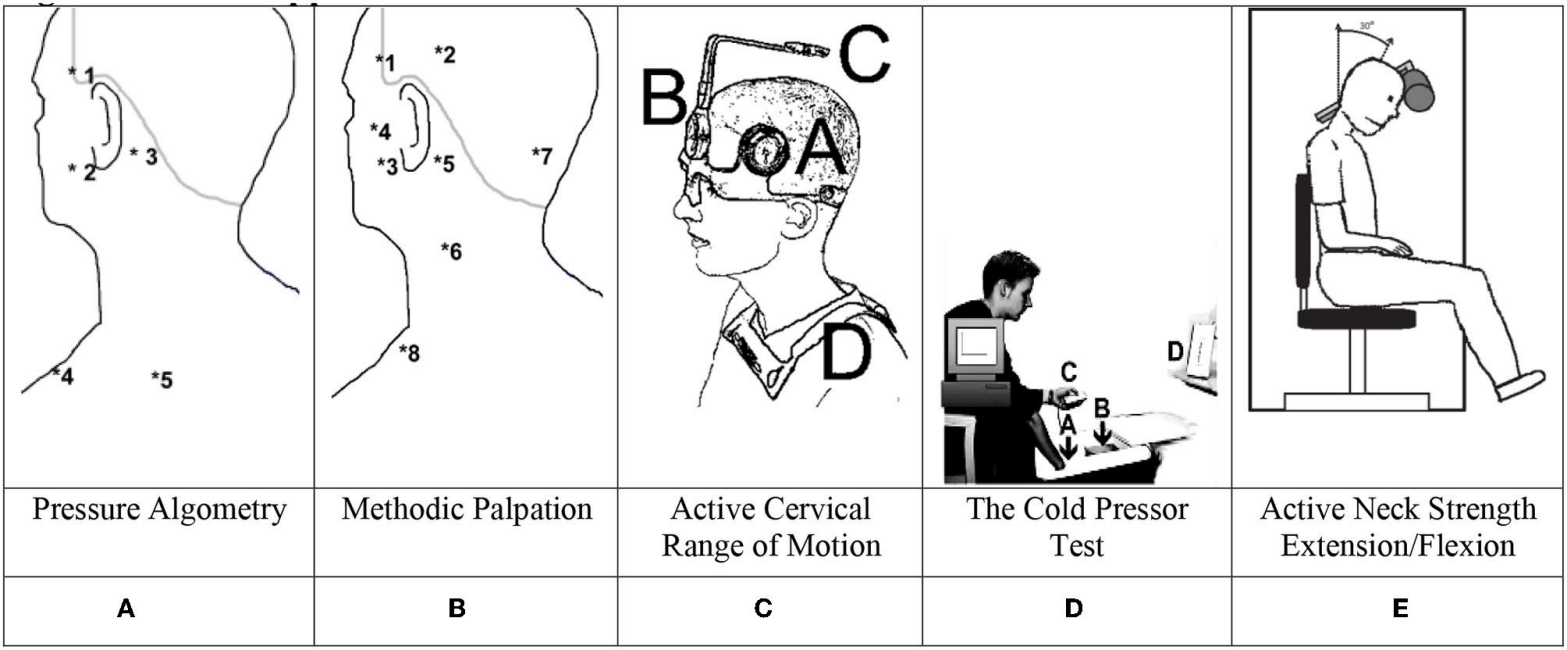
Methods applied. **(A)** Pressure algometry. **(B)** Methodic palpation. **(C)** Active cervical range of motion. **(D)** The cold pressor test. **(E)** Active neck strength extension/flexion.

A pressure algometer (SOMEDIC AB, algometer type 1) (10) was applied at trigeminal nerve-innervated muscles: (1) the temporal muscle, (2) the masseter muscle; and neck muscles at (3) the proximal insertion of the sternocleidomastoid muscle, (4) the superior part of the trapezius muscle, and (5) the infraspinatus muscle. Furthermore, the left, dorsal, proximal interphalangeal joints of the third finger was chosen as a control site for the determination of the distant pressure pain detection threshold (distant PPDT) (11). Subjects were informed to push a button when the sensation changed from a sensation of pressure to the first sensation of pain (PPDT = pressure pain detection threshold) in triplets and at five sites on the left and right sides (Figure 1A). Three different measures were computed: total PPDT, the sum of mean scores of triplets in all 10 muscles; trigeminal muscle PPDT, the sum of mean scores of four jaw muscles and the sum of mean score of six neck muscles; and distant PPDT score (mean score of left PIP).

### Methodic Palpation

Manual palpation (Figure 1B) was used to examine eight peri-cranial muscle pairs on both sides (12). The examiners used the second and third fingers to provide firm pressure on the examined muscle while making small rotational movements: 0 = no visible reaction and denial of tenderness; 1 = visible reaction but no verbal report of discomfort or mild pain; 2 = verbal report of painful tenderness with a facial expression of discomfort; 3 = marked grimacing or withdrawal with a verbal report of marked painful tenderness and pain. The tenderness score was calculated from scores of each of the 16 examined spots (TTS range 0:48). The examination order was as follows: the posterior temporal muscle (2), the anterior temporal muscle (1), the masseter muscle (3), the lateral pterygoid muscle (4) (the subject slightly opens his mouth during this procedure), the sternocleidomastoid muscle at the mastoid process (5), the sternocleidomastoid muscle at medial part (pinching) (6), the muscles inserting on superior and inferior nuchal lines (7), and the superior part of the trapezius muscle (8). The muscle pairs were simultaneously examined on the right and left sides.

The total palpation score was computed based on the sum of all 16 scores. Palpation sites 1–4 (left+right) included the eight trigeminal nerve innervated muscles during palpation and sites 5–8 (left+right) included eight neck muscles.

### Active Cervical Range of Motion

With the subject situated in a comfortable chair, the assessment of maximal voluntary flexion and extension of the neck as well as left/right rotation and left/right lateral flexion took place (total CROM was the sum of all 6 directions: flexion + extension + left lateral flexion + right lateral flexion + left rotation + right rotation), Figure 1C shows the mounted CROM instrument with A and B being the goniometers, C being a compass, and D being magnetic yoke pointing toward an artificial North pole (13–15).

### Cold Pressor Test

On each of the four examination days, the participants underwent a cold pressor test (CPT). If a participant withdrew the hand during the recording period, VAS was assigned a value of 10 from the time of withdrawal and the remaining recording period. The discomfort was immediately scored on a separate VAS scale after the hand was removed from the cold water. As shown in Figure 1D, participants immersed their dominant hand into the ice cube-free water chamber, while using the non-dominant hand to maneuver an electronic visual analog scale (e-VAS: 0 = no pain; 10 = max. possible pain), with continuous measurements during a 120-s sampling time by a computer at a frequency of 20 Hz. The CPT was carried out using an insulated box with two chambers mounted with a diffusible stainless-steel fence for separation of the two chambers. One chamber contained a water pump and 12 liters of ice cubes and 15 liters of cold tap water filled the system through the first chamber. The second chamber contained only cold water, and using the pump, the system temperature was kept at an equilibrium of 2 ± 1 °C after 15 min and during the following 60 min (16).

### Counter Stimulation

At 3 and 6 months and 1 year post-injury, an additional counter-stimulation test was applied, using both the pressure algometer as a local experimental pain source and the CPT as a general pain conditioner. The examinations took place 5 min after performing the CPT (16, 17). The pressure algometer was used with a constant slope of the indentation rate of 30 kPa/sec on the right masseter muscle, 1½ cm ante-superiorly to the mandibular angle. The participant was instructed to press a button when reaching the pressure pain tolerance threshold (PPT) at the right masseter muscle before and 15 s after the immersion of the dominant hand into cold water.

### Neck Strength and Endurance

Neck Exercise Unit, “Follo Norway,” a neck-trainer instrument (Figure 1E) with a computerized device for measuring maximal torque (Nm) was applied to examine the isometric neck muscle strength during neck extension (at 15°) and flexion (at 30°) (18, 19). First, to avoid trunk movement, the subject was restrained to the chair with a strap across the chest. To familiarize the subject with the neck movement, the intended movement was performed against a small load (1–2 kg) a few times. Finally, to establish maximal muscle strength, three maximal voluntary contractions (MVCs) were performed, and the highest obtained value was used for further analysis. The participants were instructed to “press their head against the pad as forcefully as possible” in the intended direction. Each contraction lasted approximately 10 s, during which vigorous verbal encouragement was given, and precisely 30 s of rest between each maximal effort contraction was allowed.

### Indicators of Non-Recovery

Work capacity (20) was determined after 1 year using a semi-structured interview. In the present study, the outcome was used as a binary variable, rating patients as either recovered or non-recovered persons with WP and AC were asked to select one of the six items after 1 year: (a) My work capacity is the same as before the injury, (b) I work the same number of hours as before the injury, but my tasks have been simplified or reduced due to problems encountered after the injury, (c) I have reduced my working hours and reduced work capacity due to problems after the injury, (d) I have been dismissed from my job or have changed job due to problems after the injury, (e) I am in job training due to problems after the injury, and (f) I have applied for or have received disability pension due to problems after the injury. Non-recovery was assigned to participants who selected items c–f.

### Statistics

STATA/BE 17.0 (Texas, US) was applied. A mixed design (Stata: Mixed-effects ML Regression) with two groups (WP, AC) and three groups (recovered WPs, N-WPs, and AC) was applied. The following statistics are provided using the Wald test and with degrees of freedom, number of observations, *X*^2^ values, and *p*-values. For subanalyses in the ML regression, *z*-values and *p*-values are provided with coefficients mean ± SE.

*A priori*, to find the best model fit, Akaike's information criterion (AIC) and Bayesian information criterion (BIC) were applied in the analysis of data. The use of an unstructured matrix structure provided the lowest AIC and BIC values, and this model was chosen as the preferred mixed model. Data are also presented in the margins plot. Examination days are designated 1 = 1 week; 2 = 3 months; 3 = 6 months; 4 = 1 year. *P*-values below 0.05 were considered significant. A command-line example of three groups' mixed model using global ongoing VAS_0−10_ is as follows: **vas i.recover##examday ||id:, nocons residuals(un, t(examday)) nolog**. Based on the command line, margins and margins plots were computed.

### Ethics

This study was approved by a local ethics review board (Aarhus, County Ethical Committee #1996/3799) and conformed to the Declaration of Helsinki II.

## Results

### Descriptive Characteristics

A total of 141 (74 women and 67 men) subjects with acute whiplash-injuries exposed to rear-end MVA and fulfilling the WAD I-II criteria (21) and 40 acute non-sport ankle-injured controls being pairwise matched with 40 randomly selected WPs were enrolled for the clinical prospective follow-up [Table 1 (See (8) for demographic details). Five WPs dropped out before 6 months' observation and four after 6 months; a 1-year follow-up on recovery was thus obtained for 132 WPs (8, 22). One-year non-recovery was only encountered in the whiplash group: four had changed/reduced job functioning and seven were work disabled/sick-listed].

**Table 1:** Demographics of acutely injured participants at the first visit one week post-injury.

**Injury Groups**	**Acute whiplash injury** ***n*** **=** **141** **F** **=** **74; M** **=** **67**		**Non-sport ankle injury** ***n*** **=** **40** **F** **=** **21; M** **=** **19**	
**Age (Mean ± SD)**	35.6 ± 10.8 years		34.8 ± 12.0 years	
	***n***	**%**	***n***	**%**
**Employment status**				
Employed before accident	132	93.6	35	87.5
Unemployed before accident	6	4.3	4	10.0
On leave	1	0.7	1	2.5
Retired	2	1.4	0	0.0
**Marital status**				
Single	37	26.2	17	42.5
Married/common law	100	70.9	22	55.0
Divorced	1	0.7	1	2.5
Unknown	3	2.1	0	0.0
**Educational status**				
Primary school	5	3.7	2	5.3
Secondary school	20	14.7	3	7.9
Craft training	78	57.4	18	47.4
University graduate	16	11.8	5	13.2
University incomplete	13	9.6	9	23.7
Other educational status	4	2.9	1	2.6

Initial standardized neurological examination did not reveal clinically significant findings (8) in WPs and ACs nor did the N-WPs present with specific initial neurological findings.

### Self-Reported Pain

**Figure 2:**
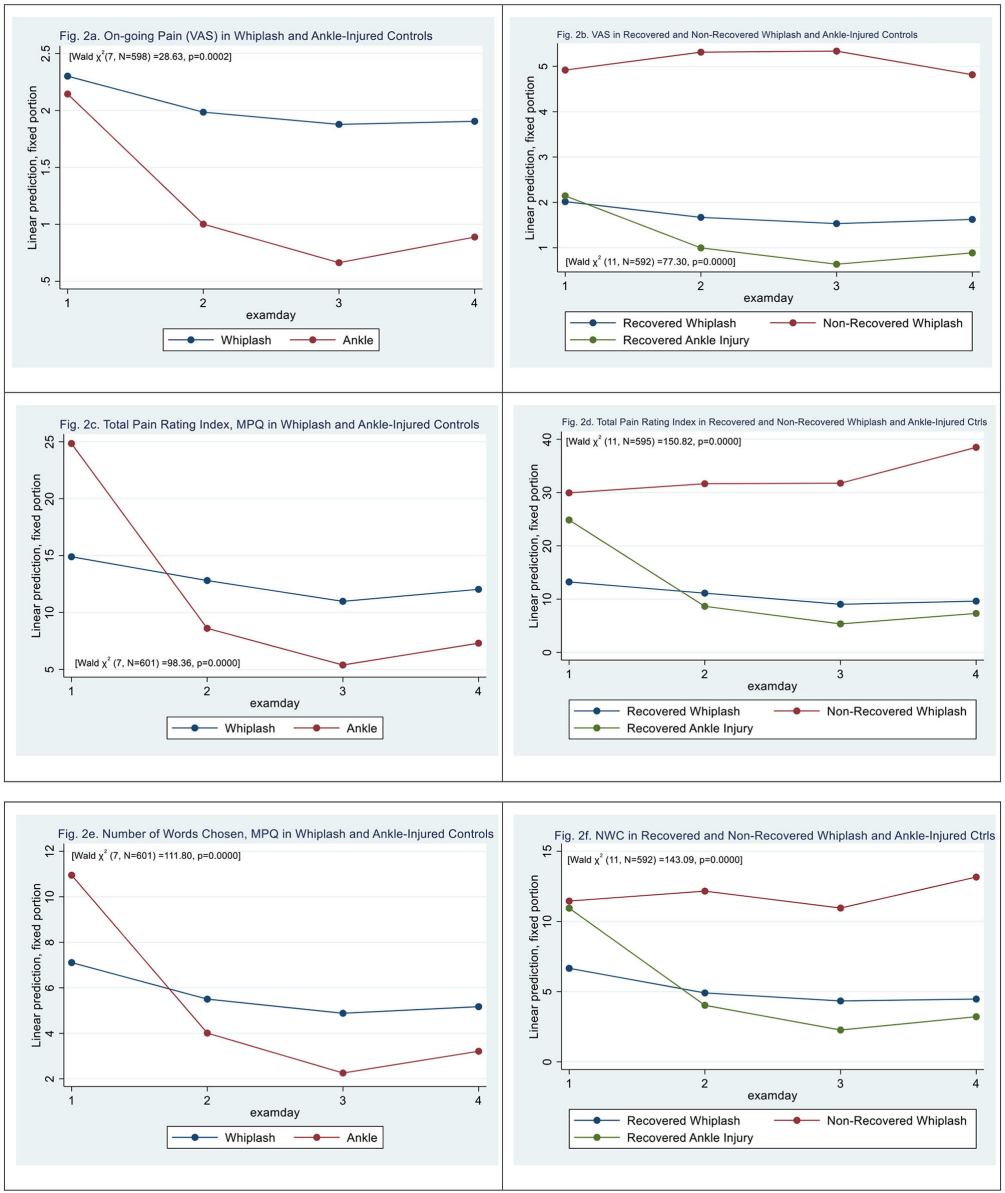
**(a)** On-going pain (VAS) in whiplash and ankle-injured controls. **(b)** VAS in recovered and non-recovered whiplash and ankle-injured controls. **(c)** Total pain rating index, MPQ in whiplash and ankle-injured controls. **(d)** Total pain rating index in recovered and non-recovered whiplash and ankle-injured controls. **(e)** Number of words chosen, MPQ in whiplash and ankle-injured controls. **(f)** NWC in recovered and non-recovered whiplash and ankle-injured controls.

Mixed-effects ML regression on ongoing self-reported pain (VAS_0−10_) revealed a significant interaction [Wald *X*^2^ (7, *N* = 598) = 28.63, *p* = 0.0002]; however, there was no significant difference between WPs and ACs, (|z| = 0.43, *p* > 0.67). There was a significant effect on examination days 3 and 4 (|z| > 2.03, *p* < 0.043) (see Figure 2a).

N-WPs were significantly different from recovered WPs [Wald *X*^2^ (11, *N* = 592) = 77.30, *p* = 0.0000], (|z| > 4.84, *p* < 0.000) with a VAS score 2.9 ± 0.59 higher than recovered. There was a significant effect on examination days 3 and 4 (|z| > 1.96, *p* < 0.05) (see Figure 2b).

The total pain rating index (PRI-T) differed between WPs and ACs [Wald *X*^2^ (7, *N* = 601) = 98.36, *p* = 0.0000], (|z| = 4.15, *p* < 0.000) with significantly higher scores at examination day 1 in ACs (|z| = 11.73, *p* < 0.000). There was an effect on examination days 3 and 4 (|z| > 2.22, *p* < 0.026) and a significant interaction between examination day and patient type (|z| > 5.04, *p* < 0.000) with higher PRI-T scores in WPs at later examinations. (see Figure 2c) N-WPs had [Wald *X*^2^ (11, *N* = 595) = 150.82, *p* = 0.0000], (|z| = 4.23, *p* < 0.000) significantly higher scores on PRI-T than recovered WPs. There was an effect on examination days 3 and 4 (|z| > 2.77, *p* < 0.006) (see Figure 2d).

Number-of-words-chosen (NWC, McGill Pain Questionnaire) (see Figure 2e) differed between WPs and ACs [Wald *X*^2^ (7, *N* = 601) = 111.80, *p* = 0.0000], (|z| = 4.21, *p* < 0.000), and there was a significant effect on all examination days (|z| > 3.24, *p* < 0.001) and a significant interaction between examination day and patient type (|z| > 4.89, *p* < 0.000). The initial score was higher in the AC group (|z| > 16.60, *p* < 0.000).

N-WPs had significantly higher NWC than recovered WPs [Wald *X*^2^ (11, *N* = 595) = 143.09, *p* = 0.0000] (|z| = 3.09, *p* < 0.002) (see Figure 2f). There was an effect on all examination days (|z| > 3.38, *p* < 0.001), and a significant interaction between examination day 4 and N-WP patient type (|z| > 2.05, *p* < 0.040).

### Pressure Algometry

**Figure 3:**
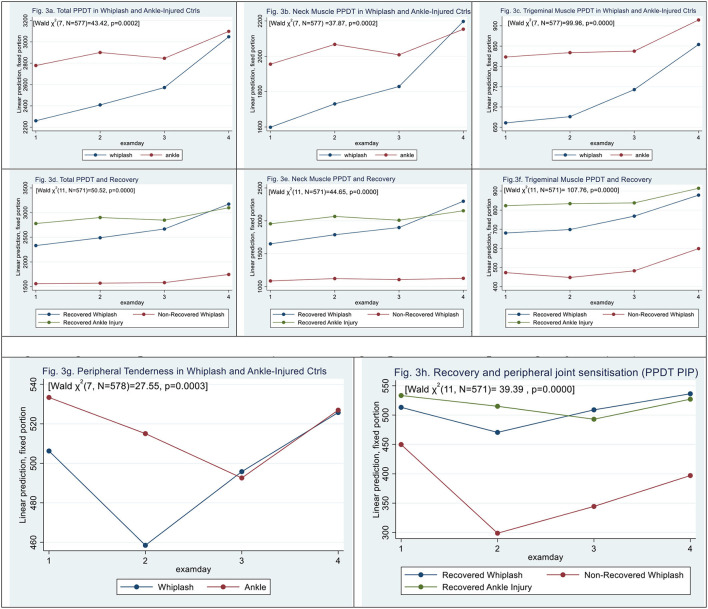
**(a–f)** Muscle tenderness assessed by pressure algometry. **(g,h)** Peripheral Tenderness [left third finger, proximal interphalangeal joint (PIP)]. **(a)** Total PPDT in whiplash and ankle-injured controls. **(b)** Neck muscle PPDT in whiplash and ankle-injured controls. **(c)** Trigeminal muscle PPDT in whiplash and ankle-injured controls. **(d)** Total PPDT and recovery. **(e)** Neck muscle PPDT and recovery. **(f)** Trigeminal muscle PPDT and recovery. **(g,h)** Peripheral tenderness (left third finger, proximal interphalangeal joint (PIP). **(g)** Peripheral tenderness in whiplash and ankle-injured controls. **(h)** Recovery and peripheral joint sensitization (PPDT PIP).

Total pressure pain detection threshold (total PPDT) was significantly lowered in WPs [mean difference 517.7 ± 218.5 kPa, (|z| = 2.37, *p* < 0.018)] when compared with ACs [Wald *X*^2^ (7, *N* = 577) = 43.42, *p* = 0.0000], with a significant effect of all examination days (|z| > 2.8, *p* < 0.005) (see Figure 3a).

Similar findings of lowered tenderness in WPs when compared with ACs were assessed by pressure algometry in the subgroups of neck muscles [Wald *X*^2^ (7, *N* = 577) = 37.87], *p* = 0.0000], with a lower threshold in WPs of 356,51 ± 162.77 kPa (|z| = 2.19, *p* = 0.028) when compared with ACs (see Figure 3b).

In trigeminal nerve-derived muscle groups [Wald *X*^2^ (7, *N* = 577) = 99.96], PPDT was lowered in WPs to 356, 51 ± 162.77kPa (|z| = 2.64, *p* = 0.008) when compared with ACs (see Figure 3c).

Examining the three recovery groups (Figure 3d) in total PPDT, there was a significant interaction [Wald *X*^2^ (11, *N* = 571) = 110.28, *p* = 0.0000]; however, N-WPs did show a significant difference from recovered WPs (|z| = 1.95, *p* = 0.052), and examination days 3 and 4 had a positive effect on scores (|z| > 2.14, *p* < 0.032).

For neck pain PPDT (Figure 3e), N-WPs was significantly lowered (|z| = 2.00, *p* = 0.046), and there was a significant effect on examination days 2, 3, and 4 (|z| > 3.37, *p* < 0.001), [Wald *X*^2^ (11, *N* = 571) = 44.65, *p* = 0.0000].

For trigeminal nerve-derived muscle groups (Figure 3f), there was a significant interaction [Wald *X*^2^ (11, *N* = 571) = 107.76, *p* = 0.0000]. N-WPs did, however, not show a significant difference from recovered WPs, (|z| = 1.93, *p* = 0.054), examination days 3 and 4 had a positive effect on scores (|z| > 4.29, *p* < 0.000).

Peripheral PPDT was assessed at the left PIP-3 joint [Wald ?^2^ (7, *N* = 578) 27.55, *p* = 0.0003]. The difference was based on contributions from examination days (|z| = 0.67, *p* > 0.5). (|z| = 2.80, *p* < 0.005); we found no significant difference between WPs and ACs (see Figure 3g), This was similarly found in N-WPs [Wald ?^2^ (11, *N* = 572 39.39, *p* < 0.0000], where the difference was based on contributions from examination days (|z| = 2.41, *p* < 0.016) (Figure 3h); we found no significant contribution from N-WPs (|z| = 0.89, *p* = 0.38).

### Palpation

**Figure 4:**
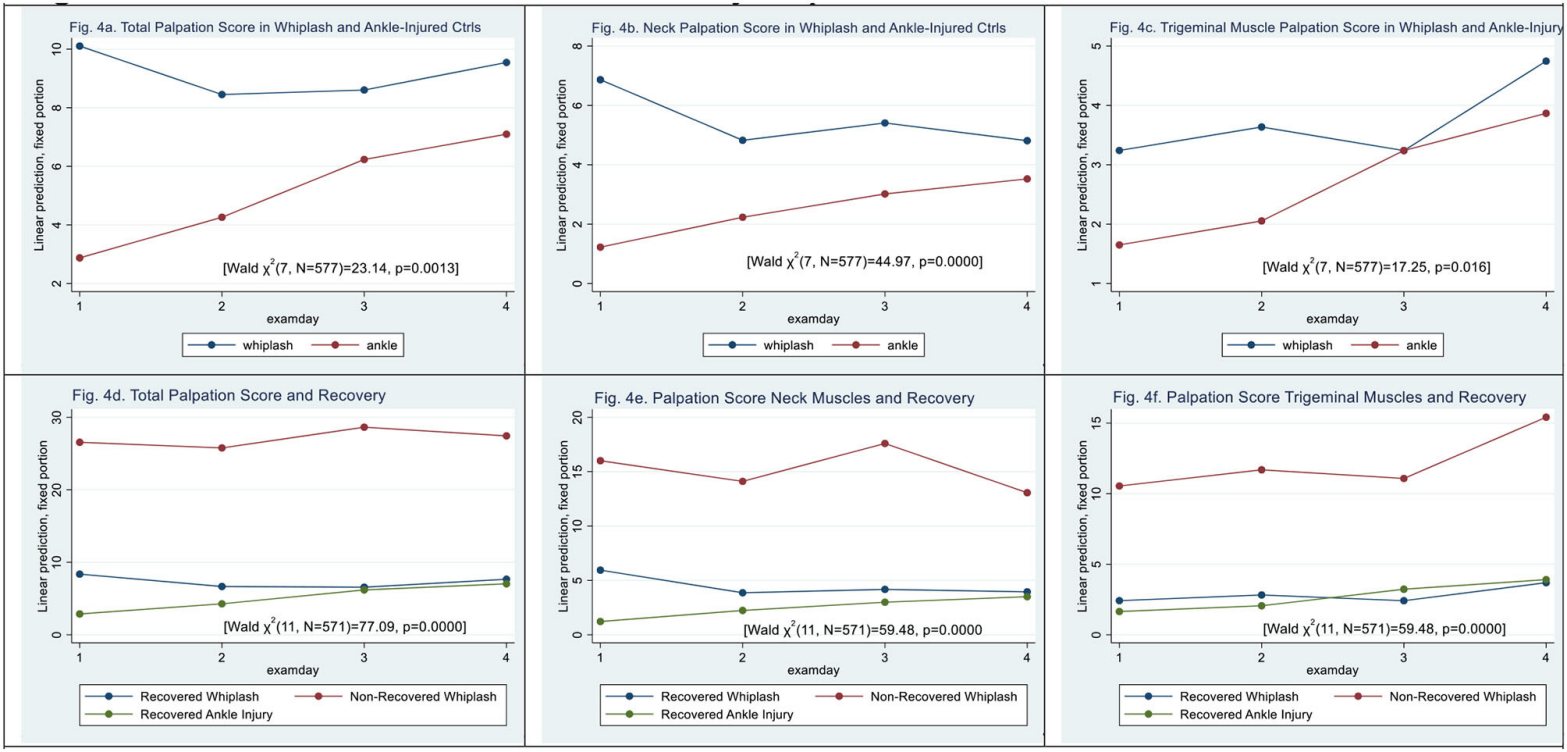
**(a–f)** Muscle tenderness assessed by palpation. **(a)** Total palpation score in whiplash and ankle-injured controls. **(b)** Neck palpation score in whiplash and ankle-injured controls. **(c)** Trigeminal muscle palpation score in whiplash and ankle-injury. **(d)** Total palpation score and recovery. **(e)** Palpation score neck muscles and recovery. **(f)** Palpation score trigeminal muscles and recovery.

Total palpation scores were significantly higher in WPs than in ACs [Wald *X*^2^ (7, *N* = 577) = 23.64, *p* = 0.0013]. Patient type (|z| = 3.75, *p* < 0.000) and examination day 2 (|z| = 4.79, *p* < 0.000) but not examination days 3 and 4 contributed to the difference. There was a significant interaction between patient type and examination days (|z| > 2.08, *p* < 0.037) (see Figure 4a).

In the palpation score of neck-derived muscles (see Figure 4b) [Wald *X*^2^ (7, *N* = 577) = 44.97, *p* < 0.000], there were significantly higher scores in WPs than ACs (|z| = 4.81, *p* < 0.000), and all examination days contributed to the difference (|z| > 2.57, *p* < 0.01). There was a significant interaction between patient type and all examination days (|z| > 2.60, *p* < 0.001).

In palpation of trigeminal-derived muscles (see Figure 4c) [Wald *X*^2^ (7, *N* = 577) = 17.25, *p* < 0.0158], patient type (|z| = 1.72, *p* = 0.086) did not significantly contribute and only examination day 4 differed significantly (|z| = 2.67, *p* < 0.007). There was no significant interaction between the patient type and the examination day (|z| <1.69, *p* > 0.092).

Regarding non-recovery, total palpation scores for examining muscle tenderness using methodic palpation scores were significantly higher in N-WPs than in recovered WPs [Wald *X*^2^ (11, *N* = 571) = 77.09, *p* < 0.0000] (see Figure 4d). Patient type N-WPs (|z| = 6.05, *p* < 0.000) contributed significantly, as did examination days 2 and 3 (|z| > 2.11, *p* < 0.035).

For neck muscles, palpation scores were raised in N-WPs when compared with recovered WPs (|z| = 5.37, *p* < 0.000) [Wald *X*^2^ (11, *N* = 571) = 97.84, *p* < 0.0000] (see Figure 4e). All examination days contributed significantly (|z| > 3.03, *p* < 0.002).

In palpation of trigeminal-derived muscles, higher palpation scores were obtained in N-WPs when compared with recovered WPs (|z| = 5.63, *p* < 0.000) [Wald *X*^2^ (11, *N* = 571) = 59.48, *p* < 0.0000] (see Figure 4f). Examination day 4 (|z| = 2.20, *p* < 0.028) contributed significantly.

### Cold Pressor Pain

**Figure 5:**
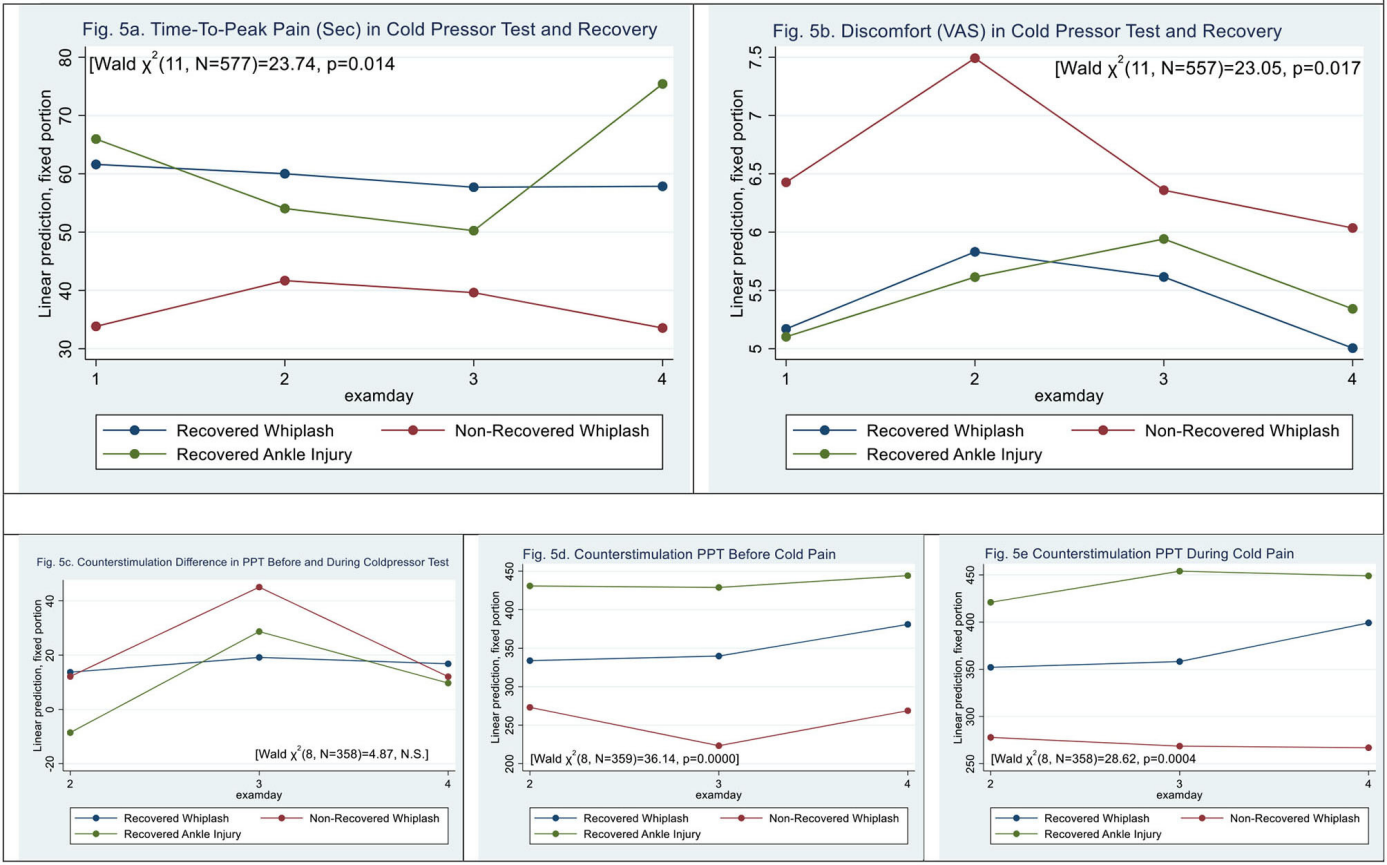
**(a,b)** Cold pressor pain. time-to-peak pain and post-exposure discomfort (VAS_0−10_). **(c–e)** Counter stimulation and recovery. **(a)** Time-to-peak pain (sec) in cold pressor test and recovery. **(b)** Discomfort (VAS) in cold pressor test and recovery. **(c–e)** Counter stimulation and recovery. **(c)** Counterstimulation difference in PPT before and during coldpressor test. **(d)** Counterstimulation PPT before cold pain. **(e)** Counterstimulation PPT during cold pain.

When examining cold pressor pain, time-to-peak pain (sec) scores were similar in WPs and ACs (|z| = 1.26, *p* > 0.2) (see Figure 5a) [Wald *X*^2^ (7, 562) = 14.66, *p* < 0.0406]. Discomfort on the VAS_0−10_ scale was similar in WPs and ACs (|z| = 0.33, *p* > 0.74).

N-WPs (see Figure 5b) had significantly shorter time to peak pain than recovered WPs and ACs [Wald *X*^2^ (11, *N* = 556) = 23.73, *p* < 0.0139], (|z| = −2.96, *p* < 0.003). The was no significant difference between N-WPs and recovered WPs (|z| = 1.34, *p* > 0.18).

### Counter Stimulation

Measures of PPT before showed a difference between WPs and ACs [Wald *X*^2^ (5, *N* = 359) = 28.73, *p* < 0.000], (|z| = 3.37, *p* < 0.001); WPs had PPT scores 102 ± 30 kPa lower than ACs before the CPT, and examination day 4 was different (|z| = 3.49, *p* < 0.000).

During the PPT during immersion of hand in cold water, there was a significant difference between WPs and ACs [Wald *X*^2^ (5, *N* = 359) = 23.38, *p* < 0.0003] (|z| = 2.25, *p* < 0.025), and examination day 4 was different (|z| = 2.99, *p* < 0.003).

No difference in PPT between before and during CPT was found [Wald *X*^2^ (5, *N* = 359) = 3.98, *p* > 0.55].

Figures 5c–e shows recovery and counter stimulation. There was an interaction in PPT before and during the CPT; however, N-WPs were not significant (|z| <1.22, *p* > 0.17).

### CROM

**Figure 6:**
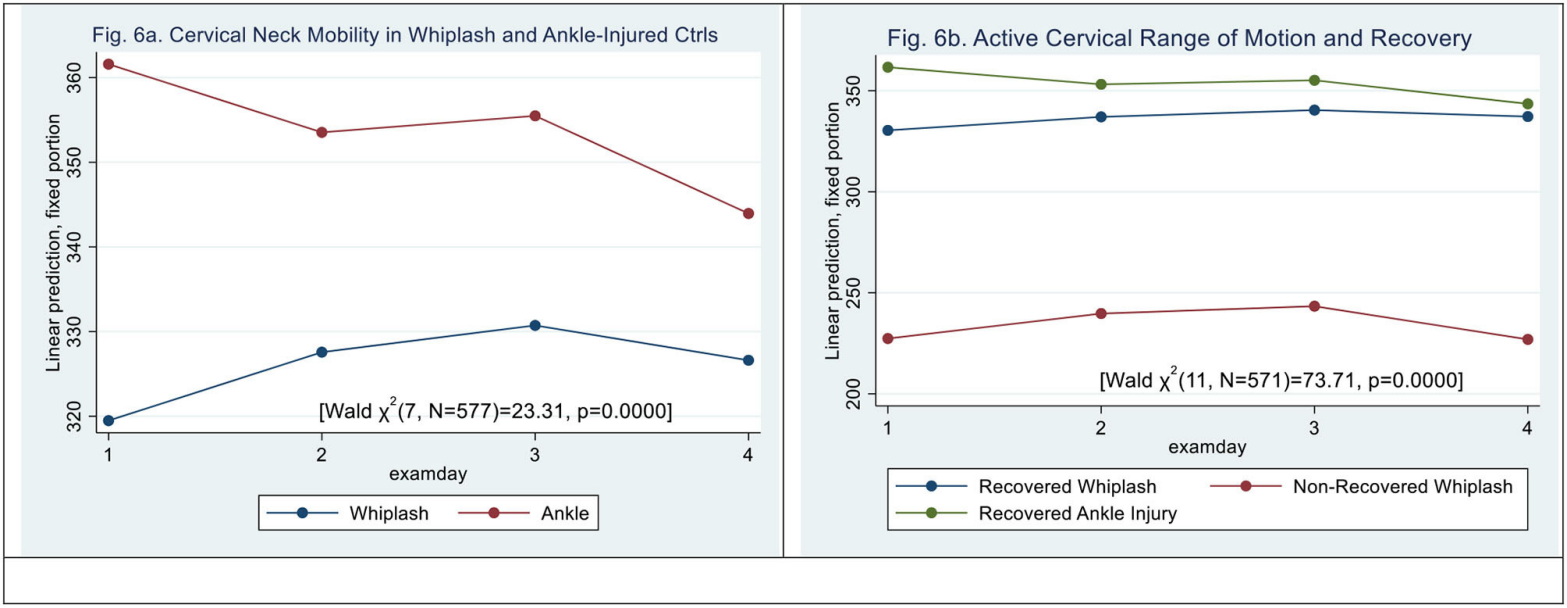
**(a,b)** Active Neck Mobility, CROM (degrees). **(a)** Cervical neck mobility in whiplash and ankle-injured controls. **(b)** Active cervical range of motion and recovery.

When examining an active cervical range of motion, mobility was significantly restricted in WPs when compared with ACs (see Figure 6a) [Wald *X*^2^ (7, 577) = 23.31, *p* < 0.0015] with a significant effect on examination days 2 and 3 (|z| > 2.08, *p* < 0.0038) but not 4 (|z| = 1.69, 0.09). There was a significant interaction between ACs and examination days 3 and 4 (|z| > 2.02, *p* < 0.04).

N-WP patient type (|z| = 6.18, *p* < 0.000) and examination day 3 did significantly contribute to the difference (|z| = 2.46, *p* < 0.014) [Wald *X*^2^ (11, *N* = 559) = 73.67, *p* = 0.0000], (see Figure 6b). However, there was no significant interaction between examination day and patient type.

### Neck Strength

**Figure 7:**
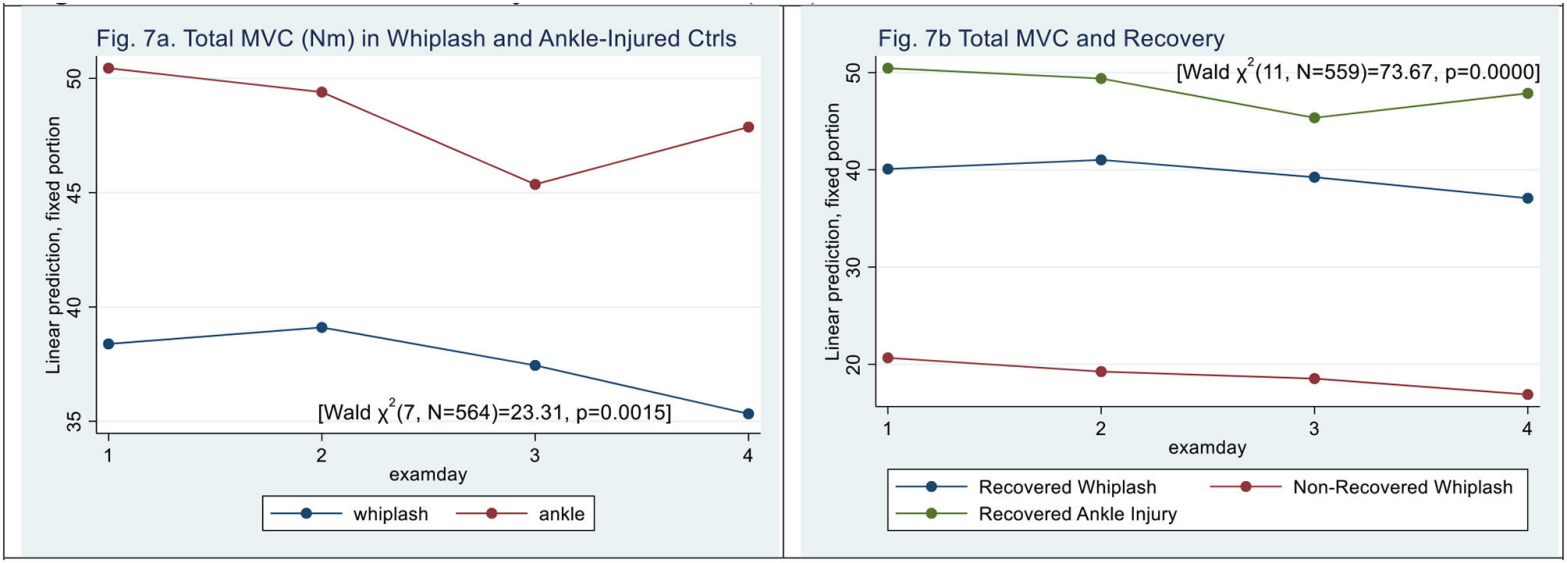
**(a,b)** Maximal Voluntary Contraction, (kPa). **(a)** Total MVC (Nm) in whiplash and ankle-injured controls. **(b)** Total MVC and recovery.

When examining maximal MVC (sum of flexion and extension, Nm), mobility was significantly restricted in WPs when compared with ACs (see Figure 7a) [Wald *X*^2^ (7, *N* = 564) = 20.13, *p* = 0.0053] with an effect of patient type (|z|= 3.04, p <0.002) and with no significant effect on examination days 2 and 3 (|z| < 0.60, *p* > 0.49) but an effect after examination day 4 (|z| = 2.00, *p* < 0.046). There was no significant interaction between ACs and examination days.

N-WPs had lower MVC [Wald *X*^2^ (11, *N* = 559) = 30.09, *p* = 0.0015] (|z| = 2.84, *p* < 0.005), and examination day did not significantly contribute (|z| < 0.5, *p* > 0.06) (see Figure 7b), and there was no significant interaction between examination day and patient type.

## Discussion

In this observational prospective study, we found initial high global pain scores in both WPs and ACs, but 1-year work-related non-recovery was only encountered in WPs. Surprisingly, the initial pain burden as assessed with NWC and total pain rating index was higher in ACs but was more abundant in WPs afterward. WPs had lowered PPDT PPT and higher palpation tenderness scores than ACs. Different patterns in muscle sensitization seemed involved in neck-related muscles and muscles confined to the trigeminal system. The use of palpation increased tenderness in N-WPs. These factors could play an important role in the clinical presentation and eventually also indicate the possibility of different treatment options in WAD if trigeminal sensitization is more involved during non-recovery, indicating an upward spreading pain (23).

Non-recovered WPs had increased pain levels at all examinations; however, remote pain was not encountered in this study (left PIP-III) and, from an early point, presented with severely affected active motor dysfunction, e.g., active neck mobility strength, local tenderness in neck and jaw, and neck-related pains that were more persistent during the 1-year observation. Cold pressor pain showed no difference between WPs and ACs but reduced time-to-peak pain time in N-WPs, indicating central sensitization in non-recovery. The CPT and time-to-peak pain were markedly reduced in the non-recovered WPs group from an early point. However, when using counter stimulation, we did not observe abnormal, aggravating, or more variating responses in non-recovered WPs than in WPs.

In more recent studies, it has been suggested that chronic WAD (I–II) is a non-specific neck pain condition, a “*de novo*” tension-type headache, as clinical features are similar to non-specific low-back pain conditions (24). Shared mechanisms for so-called spinal dyssynergia might underlie the basis for motor disability, non-recovery, and poor treatment results when applying known physical rehabilitation methods.

Cervical motor dysfunction was present in WPs but not in ACs. N-WPs experienced a marked reduction in active cervical motor function, including severely reduced active neck mobility and reduced active isometric maximal voluntary contraction, as well as correlates of central, segmental, and peripheral sensitizations of the nociceptive system.

Motor dysfunction was pronounced and more prolonged in non-recovered WPs along with long-term nociceptive sensitization in neck-related areas.

In the present study, WPs reported muscle tenderness in trigeminal nerve-innervated muscles separate from muscles anatomically innervated by upper spinal nerves and lower cranial nerves. While migraines are driven by sensitization of the trigeminal system, the upper cervical nerves (greater and minor occipital nerves) are involved in migraines, tension-type headaches, and neck pain (25). The mobility and neck strength measures are based on motor function in neck motor nerves.

Non-recovery was only encountered after whiplash injury during rear-end MVA. Motor dysfunction and early nociceptive sensitization were encountered in the non-recovered whiplash group. Within 1 week, altered pain perception and reduced motor function including both regional (neck, head, shoulder/arm) and remote/generalized dysfunction were encountered in the non-recovery whiplash group. Previously, we have reported marked changes in a larger group of high-risk WPs (26). The present findings are in line with recent studies that have found dysfunctional neuromotor control in neck-related muscles and inconsistently in the ocular muscles (27, 28) after a whiplash injury. EMG changes have been demonstrated, however, inconsistently (29–32), and similarly, inconsistently, dystonic neck muscles have been observed after an acute whiplash injury. In the present study, only active neck strength and mobility were examined.

We have previously shown that non-recovered WPs (33) report a significantly larger amount of concussion-related symptoms early after injury. After a concussion (34), a significant number of patients develop *de novo* migraine-like headaches and/or *de novo* tension-type headaches, in particular previously pain-free young persons with a mixed headache presentation.

The spinal and cerebral components after mild central neurotrauma bear great resemblance regarding cognitive dysfunction and distress (4, 35–37), but motor disabilities seem to differ between the groups, as this study also indicates.

The weakness of the present study is that it is a secondary publication reassessing previously published results. The strength of the study is the prospective observational design with a control group that was exposed to a pain-producing injury remote from the neck/head rather than a healthy control group; however, the initial matching had some limitations as it was arduous to recruit age- and sex-matched participants with AC. WPs and ACs were examined as soon as within 1 week after injury and followed up for 1 year. The main outcome was 1-year work disability (20). It could be argued that the control group was small (ACs: *n* = 40); however, the logistics and timeframe of the initial design of a prospective controlled study could not have been achieved without these limitations, given that the original study was a PhD study done in 3 years. Furthermore, the use of an examiner (HK) who was not blinded toward the diagnosis of participants with WP and AC could be inducing bias in the mere handling of the two groups. Recovery status was however determined after all examinations were done.

## Conclusion

Further investigations of initial active motor dysfunction and pain development, nociceptive sensitization, and the spread of pain in whiplash-associated disorders are still needed. The present results emphasize the complexity of neck injury exposure, thereby demonstrating the importance of including biological, psychological, and social factors in future studies (38–41). Furthermore, standardization of clinical and paraclinical measures should pave the road for future studies and hopefully bring along new treatment options after “apparently” mild neck injuries.

## Data Availability Statement

The data analyzed in this study is subject to the following licenses/restrictions: The dataset generated are available in deidentified form from the corresponding author upon request and following approval from Danish Data Protection Agency. Requests to access these datasets should be directed to helge_kasch@clin.au.dk.

## Ethics Statement

The studies involving human participants were reviewed and approved by a Local Ethics Review Board (Aarhus, County Ethical Committee #1996/3799, DENMARK). The patients/participants provided their written informed consent to participate in this study.

## Author Contributions

HK was a main part of designing and conducting the initial study, collected the quantitative data, and conducted the statistical analyses. HK and LF drafted the manuscript. HK, TC, SR, TA, and LF contributed to the design of this secondary analysis and to the formulation of the hypotheses, contributed to the interpretation and discussion of the results, and discussed the results and commented on the manuscript throughout the writing process and approved the final version. All authors contributed to the article and approved the submitted version.

## Funding

This study was supported by the Council of the Danish Victims Foundation. The initial study was funded by grants from the Danish Medical Research Council, the Danish Society of Polio and Accident Victims (PTU), the Danish Rheumatism Research Council, the Danish Pain Research Center, and Insurance & Pension Denmark. The funding agencies had no role in the data collection, analysis, interpretation, or reporting of results.

## Conflict of Interest

The authors declare that the research was conducted in the absence of any commercial or financial relationships that could be construed as a potential conflict of interest.

## Publisher's Note

All claims expressed in this article are solely those of the authors and do not necessarily represent those of their affiliated organizations, or those of the publisher, the editors and the reviewers. Any product that may be evaluated in this article, or claim that may be made by its manufacturer, is not guaranteed or endorsed by the publisher.

## References

[1] Walton DM Carroll LJ Kasch H Sterling M Verhagen AP Macdermid JC. An overview of systematic reviews on prognostic factors in neck pain: results from the International Collaboration on Neck Pain (ICON) project. Open Orthop J. (2013) 7:494–505. 10.2174/1874325001307010494 24115971 PMC3793581

[2] Sterling M Carroll LJ Kasch H Kamper SJ Stemper B. Prognosis after whiplash injury: where to from here? Discussion paper 4. Spine. (2011) 36:S330–4. 10.1097/BRS.0b013e3182388523 22020603

[3] Holm LW Carroll LJ Cassidy JD Hogg-Johnson S Cote P Guzman J. The burden and determinants of neck pain in whiplash-associated disorders after traffic collisions: results of the bone and joint decade 2000-2010 task force on neck pain and its associated disorders. J Manipulative Physiol Ther. (2009) 32:S61–9. 10.1016/j.jmpt.2008.11.011 19251076

[4] Maujean A Gullo MJ Andersen TE Ravn SL Sterling M. Post-traumatic stress symptom clusters in acute whiplash associated disorder and their prediction of chronic pain-related disability. Pain Rep. (2017) 2:e631. 10.1097/PR9.0000000000000631 29392244 PMC5741330

[5] Andersen TE Sterling M Maujean A Meredith P. Attachment insecurity as a vulnerability factor in the development of chronic whiplash associated disorder—a prospective cohort study. J Psychosom Res. (2019) 118:56–62. 10.1016/j.jpsychores.2019.01.008 30782355

[6] McLean SA. The potential contribution of stress systems to the transition to chronic whiplash-associated disorders. Spine. (2011) 36:S226–32. 10.1097/BRS.0b013e3182387fb4 22020617 PMC3232298

[7] Drewes AM Helweg-Larsen S Petersen P Brennum J Andreasen A Poulsen LH. McGill pain questionaire translated into Danish: experimental and clinical findings. Clin J Pain. (1993) 9:80–7. 10.1097/00002508-199306000-00002 8358143

[8] Kasch H Bach FW Stengaard-Pedersen K Jensen TS. Development in pain and neurologic complaints after whiplash: a 1-year prospective study. Neurology. (2003) 60:743–9. 10.1212/01.WNL.0000046661.82212.04 12629227

[9] Main CJ. Pain assessment in context: a state of the science review of the McGill pain questionnaire 40 years on. Pain. (2016) 157:1387–99. 10.1097/j.pain.0000000000000457 26713423

[10] Anonymous SOMEDIC. User's Manual for Algometer Type 1. Sweden: SOMEDIC (1991).

[11] Kasch H Stengaard-Pedersen K Arendt-Nielsen L Staehelin Jensen T. Pain thresholds and tenderness in neck and head following acute whiplash injury: a prospective study. Cephalalgia. (2001) 21:189–97. 10.1046/j.1468-2982.2001.00179.x 11442553

[12] Langemark M Olesen J. Pericranial tenderness in tension headache. Cephalalgia. (1987) 7:249–55. 10.1046/j.1468-2982.1987.0704249.x 3427625

[13] Youdas JW Carey JR Garrett TR. Reliability of measurements of cervical spine range of motion—comparison of three methods. Phys Ther. (1991) 71:98–104. 10.1093/ptj/71.2.98 1989013

[14] Youdas JW Garrett TR Suman VJ Bogard CL Hallman HO Carey JR. Normal range of motion of the cervical spine: an initial goniometric study. Phys Ther. (1992) 72:770–80. 10.1093/ptj/72.11.770 1409874

[15] Kasch H Stengaard-Pedersen K Arendt-Nielsen L Jensen TS. Headache, neck pain, and neck mobility after acute whiplash injury: a prospective study. Spine. (2001) 26:1246–51. 10.1097/00007632-200106010-00014 11389391

[16] Kasch H Qerama E Bach FW Jensen TS. Reduced cold pressor pain tolerance in non-recovered whiplash patients: a 1-year prospective study. Eur J Pain. (2005) 9:561–9. 10.1016/j.ejpain.2004.11.011 16139185

[17] Terkelsen AJ Andersen OK Hansen PO Jensen TS. Effects of heterotopic—and segmental counter-stimulation on the nociceptive withdrawal reflex in humans. Acta Physiol Scand. (2001) 172:211–7. 10.1046/j.1365-201x.2001.00856.x 11472308

[18] Jordan A Bendix T Nielsen H Hansen FR Høst D Winkel D. Intensive training, physioterapy, or manipulation for patients with chronic neck pain. Spine. (1998) 23:311–9. 10.1097/00007632-199802010-00005 9507618

[19] Krogh S Kasch H. Whiplash injury results in sustained impairments of cervical muscle function: a one-year prospective, controlled study. JRM. (2018) 50:548–55. 10.2340/16501977-2348 29767228

[20] Kasch H Bach FW Jensen TS. Handicap after acute whiplash injury: a 1-year prospective study of risk factors. Neurology. (2001) 56:1637–43. 10.1212/WNL.56.12.1637 11425927

[21] Spitzer WO Skovron ML Salmi LR Cassidy ID Duranceau J Suissa S. Scientific monograph of the Quebec task force on whiplash-associated disorders: redefining 'whiplash' and it's management. Spine. (1995) 20:1S-73. 7604354

[22] Weintraub MI Mandel S Esposito J Gordon JE Maitz EA Massari DJ. Handicap after acute whiplash injury. Neurology. (2002) 58:157–9. 10.1212/WNL.58.1.157-a 11781438

[23] Lemming D Graven-Nielsen T Sorensen J Arendt-Nielsen L Gerdle B. Widespread pain hypersensitivity and facilitated temporal summation of deep tissue pain in whiplash associated disorder: an explorative study of women. J Rehabil Med. (2012) 44:648–57. 10.2340/16501977-1006 22729792

[24] Astrup J Gyntelberg F Johansen AM Lei A Marott JL. Impaired neck motor control in chronic whiplash and tension-type headache. Acta Neurol Scand. (2021) 144:394–9. 10.1111/ane.13473 34021596

[25] Ashina S Bendtsen L Lyngberg AC Lipton RB Hajiyeva N Jensen R. Prevalence of neck pain in migraine and tension-type headache: a population study. Cephalalgia. (2015) 35:211–9. 10.1177/0333102414535110 24853166

[26] Kasch H Qerama E Kongsted A Bach FW Bendix T Jensen TS. Deep muscle pain, tender points and recovery in acute whiplash patients: a 1-year follow-up study. Pain. (2008) 140:65–73. 10.1016/j.pain.2008.07.008 18768261

[27] Fischer AJ Huygen PL Folgering HT Verhagen WI Theunissen EJ. Hyperactive VOR and hyperventilation after whiplash injury. Acta Otolaryngol Suppl. (1995) 520:49–52. 10.3109/00016489509125187 8749078

[28] Kongsted A Jorgensen LV Leboeuf-Yde C Qerama E Korsholm L Bendix T. Are altered smooth pursuit eye movements related to chronic pain and disability following whiplash injuries? A prospective trial with one-year follow-up. Clin Rehabil. (2008) 22:469–79. 10.1177/0269215507082141 18441043

[29] Falla D Jull G Hodges P Vicenzino B. An endurance-strength training regime is effective in reducing myoelectric manifestations of cervical flexor muscle fatigue in females with chronic neck pain. Clin Neurophysiol. (2006) 117:828–37. 10.1016/j.clinph.2005.12.025 16490395

[30] Jorgensen R Ris I Juhl C Falla D Juul-Kristensen B. Responsiveness of clinical tests for people with neck pain. BMC Musculoskelet Disord. (2017) 18:548. 10.1186/s12891-017-1918-1 29282073 PMC5745670

[31] Nederhand MJ Ijzerman MJ Hermens HJ Turk DC Zilvold G. Predictive value of fear avoidance in developing chronic neck pain disability: consequences for clinical decision making. Arch Phys Med Rehabil. (2004) 85:496–501. 10.1016/j.apmr.2003.06.019 15031840

[32] Nederhand MJ Hermens HJ Ijzerman MJ Groothuis KG Turk DC. The effect of fear of movement on muscle activation in post-traumatic neck pain disability. Clin J Pain. (2006) 22:519–25. 10.1097/01.ajp.0000202979.44163.da 16788337

[33] Kasch H Jensen L. Minor head injury symptoms and recovery from whiplash injury: a 1-year prospective study. Rehabil Process Outcome. (2019) 8:1179572719845634. 10.1177/1179572719845634 34497461 PMC8282153

[34] Kothari SF Eggertsen PP Frederiksen OV Møller MT Svendsen SW Tuborgh A. Characterization of persistent post-traumatic headache and management strategies in adolescents and young adults following mild traumatic brain injury. Sci Rep. (2021). 10.1038/s41598-022-05187-x 35140235 PMC8828894

[35] Carstensen TB Frostholm L Oernboel E Kongsted A Kasch H Jensen TS. Post-trauma ratings of pre-collision pain and psychological distress predict poor outcome following acute whiplash trauma: a 12-month follow-up study. Pain. (2008) 139:248–59. 10.1016/j.pain.2008.04.008 18499350

[36] Carstensen TB Frostholm L Ornbol E Kongsted A Kasch H Jensen TS. [Pre-collision pain and psychological distress predict poor outcome following acute whiplash trauma–secondary publication]. Ugeskr Laeger. (2009) 171:3431–4. 19938347

[37] Ravn SL Hartvigsen J Hansen M Sterling M Andersen TE. Do post-traumatic pain and post-traumatic stress symptomatology mutually maintain each other? A systematic review of cross-lagged studies. Pain. (2018) 159:2159–69. 10.1097/j.pain.0000000000001331 29994992

[38] Modarresi S Lukacs ML Ghodrathi M Salim S MacDermid JC Walton DM. A systematic review and synthesis of psychometric properties of the numeric pain rating scale and the visual analog scale for use in people with neck pain. Clin J Pain. (2021) 38:132–48. 10.1097/AJP.0000000000000999 34699406

[39] Griffin AR Leaver AM Arora M Walton DM Peek A Bandong AN. Clinimetric properties of self-reported disability scales for whiplash. a systematic review for the whiplash core outcome set (CATWAD). Clin J Pain. (2021) 37:766–87. 10.1097/AJP.0000000000000968 34282060

[40] Chen K Andersen T Carroll L Connelly L Cote P Curatolo M. Recommendations For Core Outcome Domain Set For Whiplash-Associated Disorders (CATWAD). Clin J Pain. (2019) 35:727–36. 10.1097/AJP.0000000000000735 31188173

[41] Bobos P MacDermid JC Nazari G Furtado R Kasch H., CATWAD. Psychometric properties of the global rating of change scales in patients with neck disorders a systematic review with meta-analysis and meta-regression. BMJ Open. (2019) 9:e033909. 10.1136/bmjopen-2019-033909 31772112 PMC6886942

